# Mitotane treatment in patients with metastatic testicular Leydig cell tumor associated with severe androgen excess

**DOI:** 10.1530/EJE-17-0542

**Published:** 2018-01-08

**Authors:** Vasileios Chortis, Nicholas J Johal, Irina Bancos, Matthew Evans, Kassiani Skordilis, Peter Guest, Michael H Cullen, Emilio Porfiri, Wiebke Arlt

**Affiliations:** 1 Institute of Metabolism and Systems ResearchUniversity of Birmingham, Birmingham, UK; 2 Centre for EndocrinologyDiabetes and Metabolism, Birmingham Health Partners, Birmingham, UK; 3 Division of EndocrinologyMetabolism and Nutrition, Mayo Clinic, Rochester, Minnesota, USA; 4 Departments of PathologyQueen Elizabeth Hospital, University Hospitals Birmingham NHS Foundation Trust, Birmingham, UK; 5 Radiology and Cancer CentreQueen Elizabeth Hospital, University Hospitals Birmingham NHS Foundation Trust, Birmingham, UK; 6 Cancer CentreQueen Elizabeth Hospital, University Hospitals Birmingham NHS Foundation Trust, Birmingham, UK

## Abstract

Mitotane (o,p′DDD) is established in the adjuvant and advanced-stage treatment of adrenocortical carcinoma and counteracts both tumor growth and tumor-related steroid production. Both the adrenal glands and the gonads are steroidogenically active organs and share a common embryogenic origin. Here, we describe the effects of mitotane in two patients with metastatic Leydig cell tumor (LCT) of the testes and associated severe androgen excess (serum testosterone 93 and 88 nmol/L, respectively; male reference range 7–27 nmol/L). Both men suffered from severe restlessness, insomnia and irritability, which they described as intolerable and disrupting normal life activities. Urinary steroid profiling by gas chromatography–mass spectrometry (GC–MS) confirmed excess androgen production and revealed concurrent overproduction of glucocorticoids and glucocorticoid precursors, which under physiological conditions are produced only by the adrenal glands but not by the gonads. In a palliative approach, they were commenced on mitotane, which achieved swift control of the hormone excess and the debilitating clinical symptoms, restoring normal quality of life. GC–MS demonstrated normalization of steroid production and decreased 5α-reductase activity, resulting in decreased androgen activation, and imaging demonstrated disease stabilization for 4–10 months. In conclusion, mitotane can be highly effective in controlling steroid excess in metastatic LCTs, with anti-tumor activity in some cases.

## Introduction

Testicular Leydig cell tumors (LCTs) are rare stromal tumors, comprising 1–3% of all testicular neoplasms (1, 2). LCTs result in precocious puberty in 10% of affected children due to excess androgen secretion (3). Affected adult men most commonly present with a painless testicular mass and significant androgen excess (4) and can also have tumor-related estrogen excess, manifesting with gynaecomastia in 10–30% of cases (4, 5, 6). An estimated 10–15% of testicular LCTs are malignant (3, 7), although the true proportion remains debated (6, 8). The primary approach to malignant LCTs is surgical, usually involving orchidectomy, retroperitoneal lymph node dissection and lifelong surveillance (9). LCT metastases are rare and are detected on average 10 years after primary surgery (7), but therapeutic options are very limited, with no known role for radiotherapy and lack of efficacy of cytotoxic chemotherapy (7, 9). Therefore, prognosis for this rare endocrine cancer is poor, with an approximate median survival of two years (3, 4, 10).

During human foetal development, gonads and adrenal glands both derive from the urogenital ridge and after separation they develop distinct steroidogenic features, with gonadal sex steroid production and adrenal production of glucocorticoids, mineralocorticoids and adrenal androgen precursors. Mitotane (o,p′DDD) is routinely used in the treatment of adrenocortical cancer, where it has been shown to control adrenal steroid excess and, to a degree, tumor proliferation (11). Mitotane also diminishes androgen action by inhibiting 5α-reductase (12) and hence activation of testosterone to 5α-dihydrotestosterone. Thus, we considered mitotane as a potentially useful drug in patients with metastatic Leydig cell tumor, in particular, in patients with tumor-associated androgen excess. Here, we describe the effects of mitotane treatment in two patients with metastatic LCT, leading to a significant biochemical and clinical amelioration of the signs and symptoms of tumor-related steroid excess, and also to temporary radiological stabilization of previously rapid disease progression.

## Methods

Urinary steroid metabolome profiling at baseline and during mitotane treatment was carried out by gas chromatography–mass spectrometry, utilizing selected-ion-monitoring analysis for identification and quantification of 32 distinct steroid metabolites reflective of 24-h net steroid output, as previously described (13). Serum steroid measurements were carried out in the routine clinical biochemistry setting, using established and validated tandem mass spectrometry (androstenedione, testosterone) and immunoassays (DHEAS, 17β-oestradiol), respectively.

We carried out immunohistochemistry for sterol-O-acyl transferase 1 (SOAT1) as described previously (14), using antibodies against SOAT1 (1:1000; ab39327; Abcam). The intensity of staining was scored as described by Sbiera and coworkers (15).

## Case reports

### Case 1

**Figure 1:**
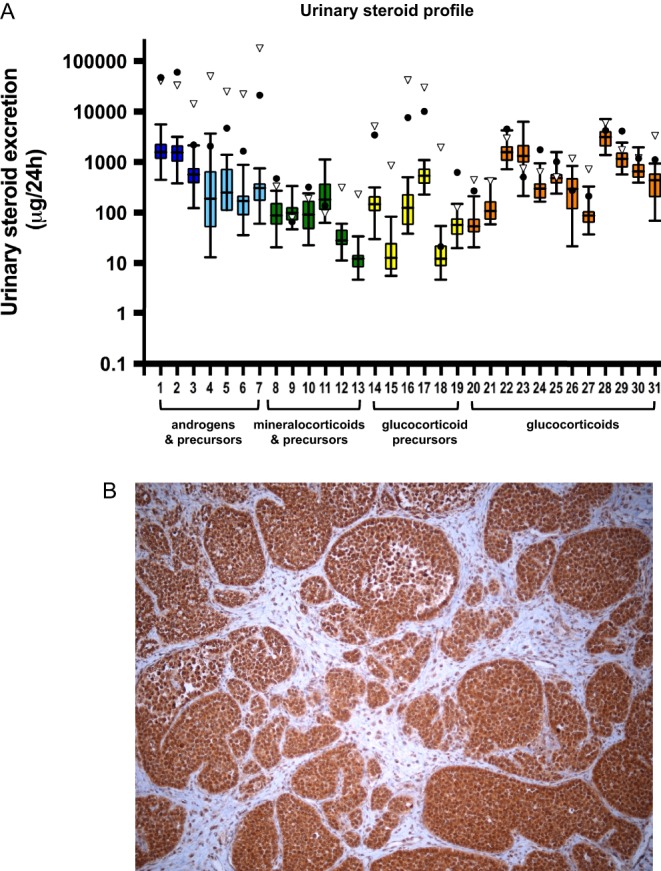
(Panel A) Steroid synthesis in the two patients with metastatic testicular Leydig cell tumor as assessed by mass spectrometry-based 24-h urinary steroid profiling before initiation of mitotane treatment (log scale; closed circles, patient 1; open triangles, patient 2). Box plots represent medians and interquartile ranges from a group of 24 healthy male volunteers (age: 40–60 years); whiskers represent the full range. (Panel B) Immunohistochemical staining for sterol-O-acyltransferase 1 (SOAT1) using formalin-fixed paraffin-embedded tissue from the recurrent tumor of patient 2, demonstrating high (60% of cells) to moderate (30% of cells) expression of SOAT1 in the tumor tissue.

A 51-year-old patient presented with severe restlessness, insomnia, impaired concentration, increased aggressiveness, redness of the face and body hair growth, all gradually developing over the last six months. Fifteen years previously, he had undergone an orchidectomy for LCT, and thirteen years later, excision of a retroperitoneal mass, confirmed on histology as LCT metastasis. Imaging revealed multiple lesions consistent with liver, lung and retroperitoneal metastases. Immunohistochemistry of a tissue biopsy confirmed vimentin-positive, inhibin-negative metastatic LCT. Serum testosterone was very high at 93 nmol/L (normal male reference range 7–27 nmol/L). Urinary steroid profiling by gas chromatography–mass spectrometry (GC–MS) showed increased androgen metabolite excretion (sum of androsterone and etiocholanolone 101,476 µg/24 h; adult male reference range <8000 µg/24 h) as well as increased excretion of DHEA, metabolites of pregnenolone, progesterone, 17-hydroxypregnenolone, 17-hydroxyprogesterone and cortisol (230 µg/24 h; normal <130) (Fig. 1A). Prognosis was assessed as poor and the patient declined chemotherapy. However, he agreed to the initiation of mitotane treatment in an attempt to improve the clinical signs and symptoms of tumor-related androgen excess that were significantly limiting his quality of life. Mitotane dose was gradually titrated to 3 g per day, with concurrent hydrocortisone replacement (20 mg tid). Within a few weeks, androgen excretion decreased from 101,476 to 12,827 µg/24 h, with evidence of significant inhibition of 5α-reductase activity and normalization of other steroids that were increased at baseline (Table 1). Plasma mitotane concentrations considered therapeutic (anti-proliferative) in the context of adrenocortical carcinoma (14–20 mg/L) (16) were reached after 5 months of treatment (Supplementary Table 1, see section on supplementary data given at the end of this article). Follow-up imaging still showed progressive disease at two months, but stable disease according to RECIST 1.1 criteria after six months of mitotane treatment (Supplementary Fig. 1). Alongside the decrease in androgens, the patient reported a significant improvement of his previously debilitating clinical signs and symptoms. He returned to full-time work and enjoyed good quality of life. After 10 months of mitotane treatment, he died suddenly of a suspected myocardial infarction; no post-mortem examination was carried out.

**Table 1:** 24-h urine steroid metabolite excretion (µg/24 h) in the two patients with metastatic Leydig cell tumor before (= baseline) and during mitotane treatment. The male reference range is derived from the 24-h urine steroid excretion observed in 24 healthy men aged 40–60 years. The numbers of the steroid metabolites relate to the numbers in Fig. 1A. The total glucocorticoid metabolites were calculated as the sum of metabolites 20, 22–25 and 27–30.

		Median (min–max) steroid excretion in healthy men (µg/24 h)	Patient 1						Patient 2	
			Baseline	Mitotane					Baseline	Mitotane
				Month 1	Month 2	Month 4	Month 6	Month 9		Month 4
	Androgen and androgen precursor metabolites									
1	Androsterone	1684 (477–5915)	44,744	13,833	11,823	9790	4448	4440	38,092	597
2	Etiocholonaolone	1668 (404–3393)	56,732	30,283	37,617	22,408	9798	8387	31,016	2697
3	11β-Hydroxy-androsterone	609 (131–2302)	2066	1414	2318	301	169	174	13,351	643
4	Dehydroepiandrosterone (DHEA)	202 (14–3948)	1939	1034	434	294	194	311	47,344	359
5	16α-Hydroxy-DHEA	269 (0–1492)	4404	9179	4098	3510	2533	6250	23,569	1126
6	5-Pregnenetriol	181 (38–951)	1558	2574	2484	2600	1575	2483	20,891	1053
7	5-Pregnenediol	326 (64–801)	19,972	27,844	24,732	25,548	9545	15,450	168,192	11,304
	Mineralocorticoids and mineralocorticoid precursor metabolites									
8	Tetrahydro-11-deoxycorticosterone	94 (22–290)	445	195	162	188	29	128	308	22
9	5α-Tetrahydro-11-deoxycorticosterone	107 (50–360)	62	100	87	52	32	49	74	18
10	Tetraydrocorticosterone	97 (24–258)	300	154	263	169	41	105	177	18
11	5α-Tetrahydrocorticosterone	193 (67–1197)	130	261	489	0	0	0	90	0
12	3α,5β-Tetrahydroaldosterone	30 (12–64)	n.m.	n.m.	n.m.	n.m.	13	28	293	25
13	Tetrahydrodeoxycorticosterone	13 (5–36)	n.m.	n.m.	n.m.	n.m.	93	343	216	37
	Glucocorticoid precursor metabolites									
14	Pregnanediol	157 (32–336)	3249	1857	1474	1171	455	646	4832	199
15	3α,5α-17-Hydroxy-pregnanolone	14 (6–89)	n.m.	n.m.	n.m.	n.m.	13	18	809	8
16	17-Hydroxypregnanolone	133 (41–537)	7163	1940	1589	1538	817	998	39,306	551
17	Pregnanetriol	576 (243–1175)	9562	5701	4295	4366	2128	2677	28,349	1495
18	Pregnanetriolone	13 (5–58)	20	2	0	3	5	0	1822	10
19	Tetrahydro-11-deoxycortisol	61 (21–159)	594	525	690	492	314	911	116	322
	Glucocorticoid metabolites									
20	Cortisol	57 (22–224)	252	735	497	495	399	813	414	201
21	6β-Hydroxy-cortisol	114 (63–504)	n.m.	n.m.	n.m.	n.m.	7657	23 193	393	3578
22	Tetrahydrocortisol	1694 (772–4534)	4260	9578	7936	5087	2115	3001	2779	1391
23	5α-Tetrahydrocortisol	1408 (229–6744)	477	344	161	114	37	66	702	40
24	α-Cortol	319 (177–1005)	1665	2566	1880	1256	524	831	597	286
25	β-Cortol	513 (255–1678)	957	378	306	207	108	153	467	60
26	11β-Hydroxy-etiocholanolone	315 (23–899)	257	147	91	95	37	50	1092	89
27	Cortisone	93 (39–348)	198	400	389	286	309	480	671	102
28	Tetrahydrocortisone	3333 (1465–7597)	3978	2391	2113	1564	763	1124	5597	807
29	α-Cortolone	1228 (605–2599)	3892	2661	2222	1166	410	539	1623	479
30	β-Cortolone	696 (417–2075)	1110	300	303	216	108	213	1130	42
31	11-Oxo-etiocholanolone	464 (74–997)	1059	734	736	868	844	1196	3144	267
Total glucocorticoid metabolite excretion		9665 (5467–15 426)	16,789	19,353	15,807	10,391	4773	7220	13,980	3408
Steroid ratios indicative of 5α-reductase										
Androsterone/etiochaolanolone		1.13 (0.05–3.00)	0.79	0.46	0.31	0.44	0.45	0.53	1.23	0.22
5α-Tetrahydrocortisol/tetrahydrocortisol		0.92 (0.05–2.27)	0.11	0.04	0.02	0.02	0.02	0.02	0.25	0.03

n.m., not measured.

### Case 2

A previously fit-and-well 59-year-old man presented with a right testicular mass and underwent orchidectomy; histopathology revealed malignant LCT. Three years later, he presented with lower back pain, and imaging showed a large retroperitoneal mass, confirmed as disease recurrence by transcutaneous biopsy. He underwent laparoscopic removal of the mass together with retroperitoneal lymph node dissection. One year later, follow-up imaging revealed disseminated metastases, including liver, kidney and peritoneal deposits. He was unwell, with agitation, anxiety and insomnia. Biochemical work-up showed increased serum testosterone (88.5 nmol/L, norma:l 7–27), oestradiol (744 pmol/L, normal <156), androstenedione (7.0 nmol/L, normal: 0.8–3.1) and DHEAS (>27 µmol/L, normal: 0.91–6.76). GC–MS profiling showed increased steroid excretion including androgen metabolites (69,108 µg/24 h, normal <8000) and cortisol (414 µg/24 h, normal <130) (Fig. 1A). He rejected chemotherapy and agreed to palliative mitotane treatment with concurrent hydrocortisone replacement; mitotane was administered employing the high-dose saturation regimen (day 1 500 mg tds, day 2 1000 mg tds, and from day 3 onwards 1500 mg tds; therapeutic plasma mitotane levels were reached after 4 months (Supplementary Table 1)). Mitotane decreased serum androgen production within four weeks. Six months after treatment initiation, plasma testosterone had decreased to 29.1 nmol/L and oestradiol to 177 pmol/L, while androstenedione and DHEAS had normalized. Urinary steroid profiling 4 months after initiation of miotane showed a decline in all previously raised steroid metabolites and decreased 5α-reductase activity. This was paralleled by significant clinical improvement in signs and symptoms, specifically reduced restlessness, aggressiveness and insomnia. Imaging four months after initiation of mitotane revealed a mixed response, with regression of some previous lesions, but emergence of new metastatic deposits in lung and abdomen. The patient passed away 12 months after his second recurrence, i.e. six months after the start of mitotane treatment.

## Discussion

Here, we used mitotane, an established drug in adrenocortical carcinoma, in two patients with metastatic testicular LCT associated with severe androgen excess, clinically manifesting with severe restlessness, insomnia, irritability and impaired concentration. Both patients experienced significant improvement in signs and symptoms with mitotane therapy, swift normalization of steroid excess and some stabilization of radiologically quantified tumor load.

In a comprehensive PubMed search (search terms: Leydig cell tumor, malignant Leydig cell tumor, metastatic Leydig cell tumor, mitotane, lysodren, and o,p′DDD), we identified eight cases of LCT treated with mitotane (Table 2). Four patients received mitotane as second- or third-line treatment for metastatic LCT for a very short time only (3 days–8 weeks); none of them showed a biochemical, clinical or radiological response. The remaining four cases received mitotane as first-line treatment for metastatic LCT, with treatment duration varying between 10 weeks and 33 months (Table 2). All four patients experienced significant radiological tumor response and reduction in steroid excess during mitotane treatment. Azer and Braunstein (17) used mitotane to treat a patient with metastatic LCT for six months, resulting in a dramatic response with complete remission of multiple pulmonary metastases, which lasted three months prior to relapse. Radiological reduction in tumor load for several months was observed in two cases (18, 19). Abelson and coworkers (20) noted a significant reduction in 17-ketosteroid excretion and clinical improvement in a metastatic LCT patient treated with mitotane for 18 months, while his disseminated metastases progressed. Adding the experience of our cases, mitotane can be considered a worthwhile palliative option in metastatic LCT, particularly when the disease is associated with steroid excess.

**Table 2:** Previously reported cases of patients with widespread metastases from testicular Leydig cell carcinoma treated with mitotane, presented in the order of duration of treatment.

Reference	Patient age (years)	Length of mitotane treatment	Mitotane dose (plasma mitotane levels)	Glucocorticoid replacement	Documented steroid excess	Patient outcome	Additional information whilst on mitotane therapy
**Second- to third-line treatment (treatment duration 3 days–8 weeks)**							
(26)	64	3 days	10 g/day (not done)	Not reported	Increased urinary 17-ketosteroids, increased urinary estrogen	Died – no effect	First-line radiotherapy (40 000 rads cobalt therapy); died 3 days after commencing mitotane therapy
(4)	37	7 weeks	1.5 g/day (not done)	Not reported	Increased urinary 17-KS, increased serum testosterone, androstenedione, DHEAS	Survived another 5 years on alternative treatment (Lonidamine)	First-line therapy cisplatin; mitotane stopped after 7 weeks due to abdominal discomfort and increasing nausea
(27)	61	8 weeks	12 g/day (not done)	Not reported	Normal urinary 17-KS and 17-OHCS	Died 8 weeks after commencing mitotane	First-line therapy cisplatin/vinblastin/bleomycin; second line therapy cyclophosphamide/doxo-rubicin/vincristine); was concurrently on chemotherapy and radiotherapy
(28)	60	8 weeks	6–12 g/day (not done)	Dexamethasone 1 mg/day	Normal 17-KS and 17-OHCS; normal serum E1, E2, Aldo	Died after 8 weeks from widespread metastatic disease	2nd line therapy (1st line doxorubicin); no response to mitotane
**First-line treatment (treatment duration 10 weeks–33 months)**							
(18)	59	10 weeks	9 g/day (not done)	None	Increased serum testosterone, estradiol, aldosterone and cortisol	Died 6 months after commencing mitotane therapy	Reduction in abdominal tumor size and reduction in testosterone and estradiol to normal levels lasting 2 months. Treatment stopped on patient’s wish following sudden deterioration and increase in tumor size
(17)	63	6 months	4–14 g/day during first four weeks, followed by 2.4 g/day	Dexamethasone 1 mg/day	Normal urinary 17-KS + 17-OHCS; normal serum aldosterone, testosterone, DHEAS, cortisol	Died after deterioration and continuing metastatic spread of disease. Clinical improvement with mitotane	Complete disappearance of pulmonary metastasis and clinical improvement after 14 weeks on mitotane. 3 months later pulmonary metastasis reappeared, mitotane was stopped and chemotherapy commenced
(20)	58	18 months	10 g initially, then 4–6 g/day (not done)	Dexamethasone 0.375 mg twice daily	Increased urinary 17-KS and estrogens	Died after clinical and biochemical improvement with mitotane but radiological progression	Believed to be clinically improving, with reduction in urinary 17-ketosteroids from 1462 to 100 mg/day
(19)	56	6 months + 27 months (9 months break in between)	4–10 g/day (15–20 mg/L)	Cortisone acetate, no dose recorded	Normal urinary 17OHCS, An, Et, DHEA; normal serum T and DHEAS	Died – metastatic disease stabilized for 18 months on mitotane treatment before disease slowly deteriorated	Decrease in retroperitoneal tumor, liver lesions, ascites along with stable disease for 18 months. Once disease deteriorated mitotane dose was escalated to 10 g/day with no effect

During human fetal development, adrenals and gonads both arise from the urogenital ridge and they both develop steroidogenic capacity, albeit with distinct features, i.e. sex steroid synthesis in the gonads and glucocorticoid, mineralocorticoid and androgen precursor synthesis in the adrenal glands. Benign testicular adrenal rest tumors, which are regularly found in men with congenital adrenal hyperplasia, have been shown to display features of both adrenal and gonadal steroidogenesis (21, 22). Using mass spectrometry, we observed that our two LCT patients showed not only androgen excess, but also increased the production of glucocorticoid precursors and cortisol, without clinical signs of Cushing’s syndrome. Two previous case reports in patients with malignant LCTs have described ectopic production of steroids normally produced by the adrenal cortex, including cortisol and aldosterone (23, 24). In our two cases, both androgen excess and glucocorticoid overproduction responded well to mitotane treatment. Comprehensive steroid metabolome mapping by GC–MS has been used successfully to differentiate malignant from benign adrenocortical tumors (13). It will be useful to test in future studies whether steroid metabolome profiling would also help differentiate benign from malignant LCT and could have a role in follow-up monitoring.

Recent studies have implicated sterol-A-acyl transferase 1 (SOAT1), previously also termed ACAT-1 for acyl-coenzyme A cholesterol acyltransferase, as a target of mitotane action (14). SOAT1 is located in the endoplasmic reticulum and involved in intracellular esterification of free cholesterol. John Achermann’s group has shown that this enzyme operates downstream of SF-1 and is important for the regulation of adrenal steroidogenesis (25). A recent study (14) has provided evidence of inhibition of SOAT1 by mitotane in an adrenocortical cell model, by demonstrating an increase in free cholesterol, oxysterols and fatty acids after treatment with mitotane. We had access to formalin-fixed paraffin-embedded tissue from the tumor recurrence in patient 2 and used it for carrying out immunohistochemistry for SOAT1 (Fig. 1B), which demonstrated predominantly high and moderate expression, detected in 60% and 30% of the cells, respectively. Thus, it is likely that both the steroid-ameliorating and anti-proliferative effects of mitotane are mediated by SOAT not only in adrenocortical carcinoma but also in LCT.

Based on our current findings, the use of mitotane in the palliative treatment of metastatic LCTs of the testes appears feasible and useful, with effective control of tumor-related steroid excess and possible beneficial effects on disease progression, a viable treatment option in a rare endocrine cancer that is not responsive to cytotoxic chemotherapy or radiotherapy.

## Supplementary Material

Supporting Figure 1

Supporting Table 1

## Declaration of interest

The authors declare that there is no conflict of interest that could be perceived as prejudicing the impartiality of this case report.

## Funding

This work was supported by the European Union under the 7th Framework Program (FP7/2007–2013, grant agreement 259735, ENSAT-CANCER, to W A), the Wellcome Trust (Clinical Research Training Fellowship WT101671AIA, to V C) and the Mayo Foundation for Medical Education and Research (Mayo Scholarship, to I B).
