# Supplementary figures and images for: Mitotane treatment in patients with metastatic testicular Leydig cell tumor associated with severe androgen excess
Source: Eur J Endocrinol. 2018 Jan 8;178(3):K21–7. doi: 10.1530/EJE-17-0542 (PMC5811932; doi:10.1530/EJE-17-0542)

**A**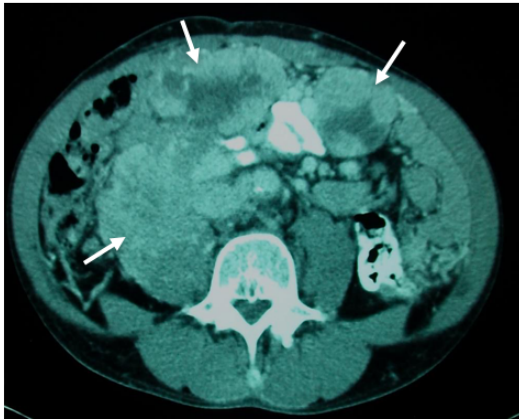**B**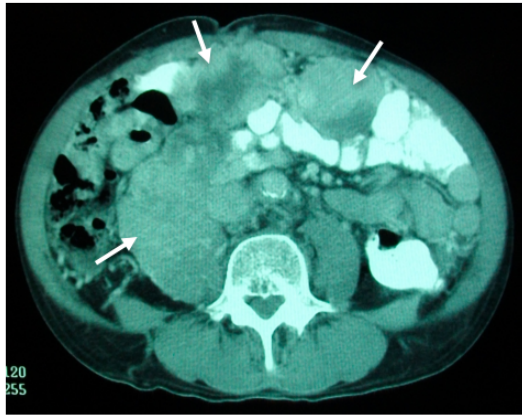

Supplement: Supporting Figure 1 [file eje-178-K21-s001.pdf]
