# Supplementary material for: Mitotane treatment in patients with metastatic testicular Leydig cell tumor associated with severe androgen excess
Source: Eur J Endocrinol. 2018 Jan 8;178(3):K21–7. doi: 10.1530/EJE-17-0542 (PMC5811932; doi:10.1530/EJE-17-0542)
Supplement: Supporting Table 1 [file eje-178-K21-t001.pdf]

Mitotane treatment in patients with metastatic testicular Leydig cell tumor associated with severe androgen excess

Suppl. Table 1: Mitotane daily dose and plasma concentrations in the two Leydig cell tumor patients. Therapeutic range for plasma mitotane in adrenal cancer is accepted as 14-20 mg/L

	Patient 1		Patient 2	
**Duration of mitotane treatment (months)**	**Mitotane Dose (g/d)**	**Plasma Mitotane (mg/L)**	**Mitotane Dose (g/d)**	**Plasma Mitotane (mg/L)**
1	1.5	4.0	4.5.	n.m.
2	3	5.9	4.5	9.1
4	3	9.1	4.5	14.2
5	3	23.5	4.5	n.m.
6	2	17.6		
7	3	9.2		

n.m., not measured.
